# Unusual Etiology of Hypercalcemia in an Adolescent With Acute Gastroenteritis

**DOI:** 10.7759/cureus.16483

**Published:** 2021-07-19

**Authors:** Madhuradhar Chrgondi, Ramya Deepthi Billa, Swathi Chacham, Shilpa Gurnurkar

**Affiliations:** 1 Pediatrics/Critical Care Medicine, Carver College of Medicine, University of Iowa, Iowa City, USA; 2 Critical Care Medicine, Stead Family Children’s Hospital, Iowa City, USA; 3 Pediatrics, All India Institute of Medical Sciences, Rishikesh, Rishikesh, IND; 4 Pediatric Endocrinology, Nemours Children's Hospital, Orlando, USA

**Keywords:** hypercalcemia, adolescent, hyperthyroidism, acute gastroenteritis, children

## Abstract

We present the report of an adolescent female who presented with acute gastroenteritis, weight loss, and hypercalcemia. Further evaluation revealed hyperthyroidism to be the cause of hypercalcemia. Treatment of hyperthyroidism successfully corrected the hypercalcemia in our index case.

## Introduction

Hypercalcemia secondary to hyperthyroidism is rare in children, unlike adults [1]. Concomitant hypercalcemic symptoms may delay the diagnosis of hyperthyroidism and it should be considered in the differential diagnosis of hypercalcemia. Here, we report an adolescent female who presented with acute gastroenteritis, weight loss, and hypercalcemia. Further evaluation revealed hyperthyroidism to be the cause of hypercalcemia. Treatment of hyperthyroidism successfully corrected hypercalcemia.

## Case presentation

A 15-year-old female with attention-deficit/hyperactivity disorder (ADHD) presented to our hospital with a four-week history of intermittent watery stools, non-bilious vomiting, and a 40-pound weight loss. Prior to admission, she was evaluated at urgent care centers on multiple occasions where she was treated with ondansetron and omeprazole with only temporary improvement in her symptoms. She was subsequently referred to our gastroenterology clinic and admitted to the medical floor for rehydration and further evaluation.

Upon reviewing the history, the patient complained of mild to moderate epigastric pain, palpitations, and dizziness. She denied fever, fainting, ingestion of drugs, or travel outside the United States. Intravenous hydration was initiated, and an initial laboratory workup was sent. She was found to have persistent tachycardia. Laboratory workup was remarkable for hypercalcemia (total serum calcium 15.3 mg/dL and ionized calcium 2.05 mmol/L) and hypernatremia (161 mEq/L). The patient was then transferred to the Pediatric Intensive Care Unit (PICU) for further management. Upon admission to the PICU, her heart rate and blood pressure were elevated at 150 beats per minute and 130/80 mm Hg, respectively. She received multiple fluid boluses, with minimal response. On physical exam, she was noted to have a diffuse goiter. Endocrinology consult was requested and further workup revealed severe hyperthyroidism with a suppressed thyroid-stimulating hormone (TSH) at <0.015 uIU/mL, an elevated total thyroxine (T4) at 16.20 mcg/dL (normal 4.50-10 mcg/dL), a very elevated free T4 greater than 6.99 ng/dL (normal 0.8-2 ng/dL) (by electrochemiluminescence immunoassay), elevated total triiodothyronine (T3) of 413 ng/dL (normal 100-210 ng/dL),with positive antithyroglobulin antibody titers at 7 IU/mL (normal 1 IU/mL), and an elevated thyroglobulin level, at 141 ng/mL (normal <40 ng/mL). Other causes of hypercalcemia were excluded. The intact parathyroid hormone (iPTH) level was normal at 9.1 pg/mL (normal 7.5- 53.5 pg/mL), thus ruling out hyperparathyroidism as a cause of hypercalcemia. Serum vitamin D level was slightly low at 22 ng/mL (normal 30-100 ng/mL), and normal alkaline phosphatase level, 130 IU/L (normal 60-134 IU/L). Urinary calcium excretion and random urine protein, creatinine ratio, were also normal. Serum levels of adrenocorticotropic hormone (ACTH), cortisol, aldosterone, and vitamin A were also normal. The patient’s white blood cell count, hemoglobin, serum phosphorus, serum magnesium, renal, and liver function tests were all normal. Urine pregnancy tests and urinary toxicology screens were negative. An oncology consult was requested to rule out malignancy as the cause of hypercalcemia. Serum lactate dehydrogenase (LDH) and erythrocyte sediment rate (ESR) were both normal. On ultrasound examination, the thyroid gland showed mild diffuse enlargement with extreme hypervascularity. A renal ultrasound revealed no nephrocalcinosis or calculi. An electrocardiogram (EKG) indicated sinus tachycardia, and her echocardiogram was normal. The patient did not have clinical symptoms or signs suggestive of a thyroid storm. She was diagnosed with thyrotoxicosis and started treatment with propranolol, methimazole, and potassium iodide to help lower her thyroid hormone levels rapidly. Hypercalcemia and hypernatremia were corrected with appropriate intravenous fluids (IVF) and furosemide. Over the next three days, her symptoms, as well as serum sodium and calcium levels improved, and she was discharged home after a one-week hospital stay. Unfortunately, she was readmitted with vomiting and significant hyperthyroidism two weeks later due to poor compliance. Initially, she was treated with potassium-iodide and propranolol and due to her medical and social situation, a decision was made to proceed with the permanent treatment of the hyperthyroidism. She underwent a total thyroidectomy. The postoperative period was uncomplicated. Her serum calcium levels normalized, and she was discharged home on levothyroxine. Additionally, treatment with calcium carbonate and calcitriol was commenced due to the expected transient hypocalcemia from a prolonged suppression of iPTH. At a follow-up office visit, she was asymptomatic with normal serum calcium levels and improving thyroid hormone levels.

## Discussion

In children, the association of hypercalcemia with hyperthyroidism is infrequent [1]. In adults, however, it is relatively common [1]. The mechanism of hypercalcemia in hyperthyroidism is not well understood. The one postulated mechanism is the modification of the bone remodeling cycle through T3-mediated osteoclastic activation [2]. In our patient, the T3 level was very elevated. Besides, the ongoing vomiting and dehydration may have also contributed to hypercalcemia [3]. In patients with serum calcium levels above 3.25 mmol/L, other causes of hypercalcemia, such as hyperparathyroidism, should be excluded [4]. Our patient had normal serum phosphorus and an appropriately low normal iPTH level secondary to the hypercalcemia associated with hyperthyroidism. The enzyme 1-alpha hydroxylase that converts 25-hydroxyvitamin D to 1,25-dihydroxyvitamin D is driven by parathyroid hormone level [5], thus explaining the low 1,25-dihydroxyvitamin D levels. Hyperthyroid patients generally have a voracious appetite [6]. Reduced appetite and vomiting in our patient at presentation were likely secondary to hypercalcemia. These symptoms can mask the classic presentation of hyperthyroidism and result in a thyroid storm by the time of diagnosis.

A thyroid storm or thyrotoxic crisis occurs due to the sudden release of the thyroid hormones T4 and T3 into the circulation and results in acute worsening of the hyperthyroid state [7]. Clinical features of thyroid storm include tachycardia, fever, agitation, dehydration, shock, congestive failure, and if not treated, death [7,8]. Our patient presented with tachycardia and dehydration but no fever, shock, or congestive failure, and was therefore not in thyroid storm on presentation. In patients with hyperthyroidism-associated hypercalcemia, the first-line therapy includes aggressive hydration with the addition of diuretics alongside treatment to normalize serum thyroid hormone levels [1]. Other treatment modalities include steroids, calcitonin, and pamidronate in refractory cases [1].

## Conclusions

Hypercalcemia secondary to hyperthyroidism is uncommon in children. In patients presenting with hypercalcemia of unknown etiology, hyperthyroidism should be considered in the initial differential. Conversely, in hyperthyroid patients presenting with atypical symptoms such as anorexia, abdominal pain, and dehydration, serum calcium levels should be checked to evaluate for hypercalcemia.
